# *Anopheles pseudowillmori *is the predominant malaria vector in Motuo County, Tibet Autonomous Region

**DOI:** 10.1186/1475-2875-8-46

**Published:** 2009-03-16

**Authors:** Wu Song, Pan Jia-Yun, Wang Xue-Zhong, Zhou Shui-Sen, Zhang Guo-Qing, Liu Qian, Tang Lin-Hua

**Affiliations:** 1 National Institute of Parasitic Diseases, Chinese Center for Disease Control and Prevention, Shanghai, PR China; 2 Yunnan Institute of Parasitic Diseases, Puer, PR China; 3 School of Integrated Traditional and Western Medicine, Anhui College of Chinese Traditional Medicine, Hefei, PR China

## Abstract

**Background:**

Malaria is endemic in Linzhi Prefecture in the Tibet Autonomous Region (TAR), but the vector for malaria transmission had never been identified.

**Methods:**

Adult *Anopheles *spp. were collected in Motuo County, Linzhi Prefecture on the Sino-Indian border in July and August, 2007. Multiplex PCR was adopted for species identification, and a nested PCR approach was used to detect sporozoites in the salivary glands of the mosquitoes.

**Results:**

3,675 mosquitoes of the *Anopheles maculatus *group were collected and processed for species identification. Among them, 3,602 (98.0%) were *Anopheles pseudowillmori *and 73 (2.0%) were *Anopheles willmori*. The *Plasmodium vivax *SSUrDNA fragment was amplified in two of 360 pooled *An. pseudowillmori *samples.

**Conclusion:**

The local *An. maculatus *group comprises the species *An. pseudowillmori *and *An. willmori*. *Anopheles pseudowillmori *is considered the sole malaria vector in Motuo County in Linzhi Prefecture.

## Background

Linzhi Prefecture is located in the south-eastern part of the Tibet Autonomous Region (TAR) of China. Motuo County in south of Linzhi Prefecture had a total population of 10,019 in 2006 and shares borders with both India and Myanmar. A total of 2,459 malaria cases were reported from Linzhi Prefecture between 1986 and 2004. Most of these infections were attributed to *Plasmodium vivax*, and 2,441 (99.3%) of the cases originated from Motuo County [1]. In 2005 and 2006, the annual malaria incidence rates (IR) in Motuo County were 56.8 and 69.4 cases per 10,000 persons, respectively. Also in 2005, a malaria outbreak was reported from Bayi Town out of Motuo County, indicating an increasing malaria threat.

Up to the present day, Motuo County is inaccessible by car, and most villages can only be reached by foot. Although malaria appears to be a considerable public health problem in Motuo County, only few studies focused on this area, and the local malaria vector(s) remained elusive. This can be attributed both to the lack of local health workers, and to the challenging geographical conditions.

Studies conducted in 2006 established that *Anopheles maculatus *s.l. is the dominant *Anopheles *taxon in Motuo County (unpublished). The *An. maculatus *Theobald group belongs to the Neocellia series of the subgenus *Cellia *and is distributed throughout the Oriental region [2]. Globally, nine members of the *An. maculatus *group have been described [3,4]. Five among them occur in China. These are *An. maculatus *s.s., *An. willmori*, *An. pseudowillmori*, *Anopheles sawadwongporni *and *Anopheles dravidicus *[5,6]. *Anopheles maculatus *s.l. and related species have long been recognized as important malaria vectors in Malaysia [7], Thailand [8] and the Philippines [9]. *Anopheles maculatus *s.l. infected with *Plasmodium *were also detected in Yunnan Province, south-west China [10].

The aim of the present study was to elucidate the significance of *An. maculatus *s.l. for malaria transmission in Motuo County, thus providing the basis for the development and implementation of a locally adapted integrated malaria control strategy.

## Methods

### Study sites and period

Motuo County stretches across the lower reaches of the Brahmaputra River between 27°36' and 29°50' N latitude and 93°42' and 96°36' E longitude. The altitude of the mountainous county ranges between 700 and 2,100 m above sea level (mean: 1,200 m) and the average annual temperature is 16.1°C. Almost all malaria-endemic villages are scattered along the Brahmaputra River. These villages are mostly inhabited by members of the Zang, Menba and Luoba nationalities.

**Figure 1:**
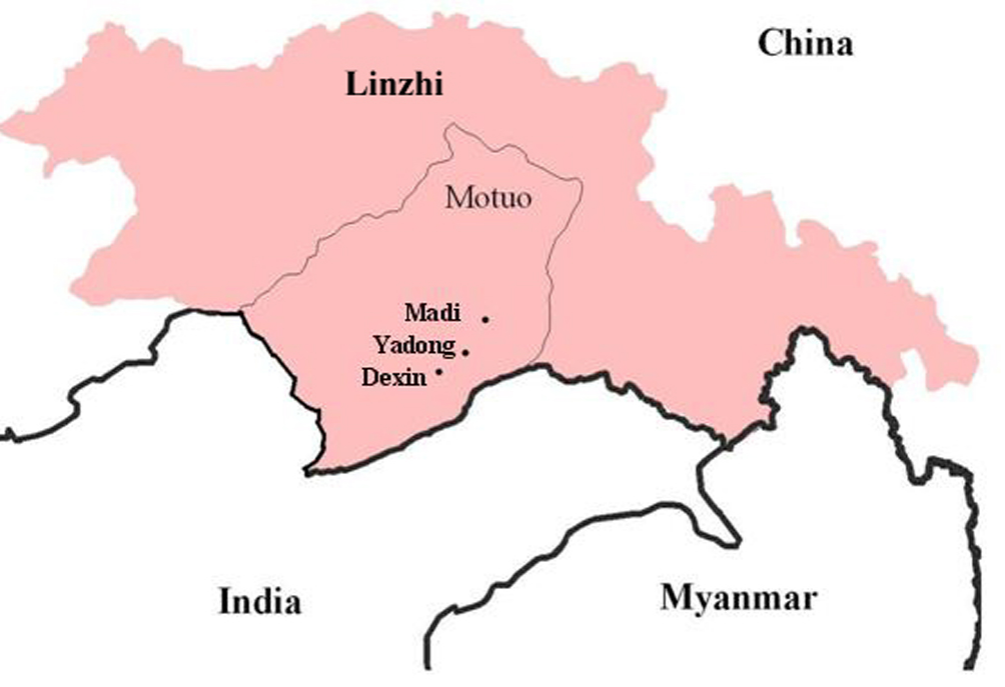
**The map of relative study sites**.

The study was implemented during the peak malaria transmission season from July to September 2007 in three villages(Figure 1) where the malaria incidence was high in previous years, namely Yadong (29°32'N, 95°33'E), Dexin (29°33'N, 95°30'E) and Madi (29°37'N, 95°41'E) near the Sino-Indian border. The inhabitants of these villages live in houses and huts grouped into hamlets scattered across the village territory, usually 1–3 km apart from each other and only accessible by foot.

### Sample collection

Cow-baited traps (CBT), human-baited net traps (HBNT) and CDC light traps (New Standard Miniature Light Traps 512 6 V 150 mm) were set up to collect adult mosquitoes between 21:00 and 01:00, or between 21:00 to 06:00. Each morning, the trapped mosquitoes were counted and identified according to morphological criteria using the key developed by Lu BL [5]. According to Manguin et al. [2], the morphological identification of adult members of the *An. maculatus *group is error-prone due to overlapping characteristics; therefore, the collected samples were morphologically classified by species complex rather than species. Following the classification, the mosquitoes were killed by chloroform and dried on silica-gel, and subsequently transported to the laboratory where they were stored at -20°C pending DNA extraction.

### DNA extraction

All *An. maculatus *s.l. adults were individually registered. Subsequently, about 70% *An. maculatus *s.l. DNA was extracted from the legs of each individual for species identification by multiplex PCR, and pooled samples each comprising 10 thoraxes of the same species were homogenized to extract DNA for sporozoites identification as described by Li et al. [11] and Sun et al. [12]. DNA extraction followed the protocol presented by Collins et al. [13], and the quality of the isolated DNA was assessed by measuring the proportional optical density (OD) at 260/280 nm.

### Species identification by multiplex PCR

3,675 *An. maculatus *s.l. specimens underwent species identification by multiplex PCR. A protocol capable of distinguishing five members of the *An. maculatus *s.l. was adapted from Ma et al. [6], with primers designed based on sequence variations in the second internal transcribed spacer (ITS2) of the ribosomal DNA (rDNA). *Anopheles pseudowillmori*, *An. maculatus *s.s., *An. willmori*, *An. dravidicus *and *An. sawadwongporni *are characterized by 119 bp, 186 bp, 231 bp, 327 bp and 406 bp fragments, respectively. Well-characterized DNA samples were used as positive controls. The amplified products were spread by electrophoresis on a 2% agarose gel, stained with ethidium bromide, and visualized using the Gel Imaging System (Alpha Imager HP, USA). A subsample of the PCR products was cloned and sequenced for confirmation.

### Detection of *P. vivax *by nested PCR

The DNA of 3,600 *An. pseudowillmori *(= 360 pooled samples) and 70 *An. willmori *(= 7 pooled samples) thoraxes was extracted according to the Collins method [13]. For the nested PCR, the following primers described by Snounou et al. [14] were used: the *Plasmodium *specific SSUrDNA primers rPLU5 (5'-CCTGTTGTTGCCTTAAACTTC-3') and rPLU6 (5'-TTAAAATTGTTGCAGTTAAAACG-3'), and the *Plasmodium vivax *species-specific SSUrDNA primers rVIV1 (5'-CGCTTCTAGCTTAATCCACATAACTGATAC-3') and rVIV2 (5'-ACTTCCAAGCCGAAGCAAAGAAAGTCCTTA-3'). DNA amplification followed the protocol of Tassanakajon [15]. Specifically, the first amplification cycle was performed using the primers rPLU5 and rPLU6 in 50 μl reaction mixture containing 1 μl DNA template. For the second cycle, 1 μl of the first-round product was used as template for the primers rVIV1 and rVIV2. Pooled samples were considered positive if a 121 bp fragment was obtained in the second PCR cycle. All products were cloned, sequenced and blasted in NCBI BLAST .

## Results

### Anopheline species complex and species identification

**Figure 2:**
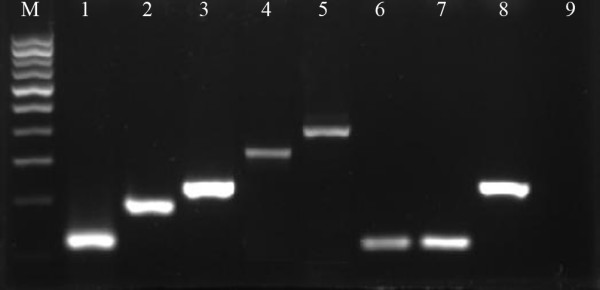
***Anopheles maculatus *s.l. species identification by PCR**. Ethidium bromide-stained PCR products from five species belonging to *An. maculatus *s.l. as well as positive samples collected in Motuo County, TAR after electrophoresis on agarose gel. Lane M: 100 bp ladder; lane 1: *An. pseudowillmori*; lane 2: *An. maculatus *s.s.; lane 3: *An. willmori*; lane 4: *An. dravidicus*; lane 5: *An. sawadwongporni*; lanes 6–8: *Anopheles *spp. collected in Motuo County, TAR; lane 9: negative control.

During the study period, a total of 5,345 adult anopheline mosquitoes were collected by HBNTs, CBTs and CDC light traps. Among them, *An. maculatus *s.l. was the predominant taxon representing 97.1% (5,190) of the total number of collected anopheline mosquitoes. *An. peditaeniatus *accounted for 2.9%(155). A total of 3,675 morphologically-identified *An. maculatus *s.l. were randomly selected for species identification by multiplex PCR. The 231 bp fragment corresponding to *An. willmori *and the 119 bp fragment associated with *An. pseudowillmori *were amplified 3,602 (98.0%) and 73 (2.0%) times, respectively (Figure 2). Cloning and sequencing of the 231 bp fragment yielded the following sequence: 5'-CTGCAGGGCACATGAACACCGTTGAACGCATATTGCGCATCGGACGCTTCAACCCGACCGATGCACACAT CCTTGAGTGCCTACCAAGTTATCTATTTTCTCCTACCAAACTGACCGTCCCATCCCCGTGATG GGCTGTCGCAGCATGGCGTGCTCGGACCCGCATCTGTCGGGACCGTGGGCGTTGATAGTGAGAGTGCTATTAT AACGAATGGGGTTACACTATGGGGC-3'. The sequence of the 119 bp fragment was: 5'-GAACTGCAGGACACATGAACACCGTTGAACGCATATTGCGCA TCGGACGATTCACCCGACCGATGCACACATCCTTGAGTGC CTACTCAGTTATCTTATATGCCCATACCAGACTAGAC-3'. The NCBI Blast revealed a homology of 98% between the 231 bp fragments and the *An. willmori*-specific sequence AF512552.1. The respective value for the 119 bp fragment and *An. pseudowillmori *sequence AF512550.1 was 97%.

### Sporozoite detection

**Figure 3:**
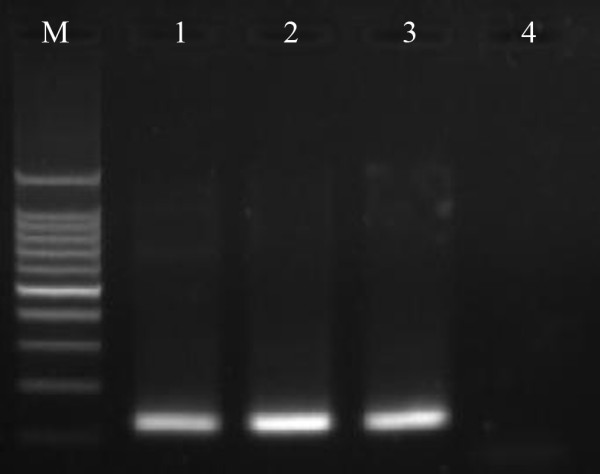
**Sporozoite detection by PCR**. Ethidium bromide-stained PCR products from the nested PCR approach for sporozoite detection. Lane M: 100 bp ladder; lane 1: positive control; lanes 2–3: pooled samples from Motuo County, TAR; lane 4: negative control.

A 121 bp fragment was amplified from two of the 360 pooled *An. pseudowillmori *samples (Figure 3). Cloning and sequencing of the product confirmed the presence of the *P. vivax *SSUrDNA fragment which was found to be identical with the previously reported sequence: 5'-ACTTCCAAGCCGAAGCAAAGAAAGTCCTTAAAAAGAATCATTTTAATTAAAAGAACACATAATAGCAAAA TGCGCACAAAGTCGATACGAAGTATCAGTTATGTGGATTAAGCTAGAAGCG-3' (AF145335, nt 520 – 640). The amplification of seven pooled *An. willmori *samples yielded no positive results.

## Discussion

This is the first investigation aiming at identifying the malaria vector(s) in Motuo County of Linzhi Prefecture, TAR. According to the present results, the *Anopheles *spp. population in Motuo County almost exclusively consists of *An. maculatus *s.l. with *Anopheles peditaeniatus *accounting for the remainder. A previous study [16] reported that *Anopheles sinensis*, one of the major malaria vectors in central China, also occurred in the TAR. However, the present study failed to consolidate this claim. Although an experimental study [17] had confirmed that the sporozoites of *Plasmodium cynomolgi *could develop in *An. peditaeniatus *and that this mosquito could also transmit them to monkeys, there currently is no direct evidence for the transmission of human malaria by this species. Thus, *An. maculatus *s.l. can be regarded as the sole local malaria vector.

The study further showed that the *An. maculatus *group in Motuo County comprises the two species *An. willmori *and *An. pseudowillmori*. *Anopheles willmori *is one of the primary malaria vectors in Nepal [18] and has previously been reported to occur in the TAR [5]. However, its local relevance for malaria transmission might be limited regarding its smaller abundance compared to *An. pseudowillmori*. The latter has been established as a malaria vector in northwest Thailand along the border with Myanmar [19,20] but has not been recorded in the TAR before. Dong et al. [21], however, reported a clear relationship between the seasonal abundance variation of *An. pseudowillmori *and malaria IR in a mountainous area of Yunnan Province in the south of the TAR.

The detection of sporozoites in the salivary glands of *Anopheles *spp. conclusively identifies them as malaria vectors. Traditionally, sporozoite infection rates were determined by dissection and examination of salivary glands of individual mosquitoes under a light microscope, a time consuming and labor intensive approach. Therefore, alternative sporozoite detection methods had been developed in recent years: enzyme-linked immunosorbent assay (ELISA) [7,9,22-24] detecting the circumsporozoite protein (CSP), and different approaches based on the PCR technique [25,26] aiming at amplifying specific DNA sequences. It appears that the detection of *Plasmodium falciparum *and *P. vivax *specific DNA sequences in mosquitoes by PCR has a higher sensitivity compared to ELISA tests [27].

In this study, a nested PCR approach aiming at the identification of the SSUrDNA of *P. vivax *sporozoites had been selected to identify malaria vectors. A conventional PCR test can detect as few as 10 sporozoites per salivary gland, making it a useful tool for screening small numbers of *Anopheles *spp. [15]. The sensitivity of the employed nested PCR test is as low as three sporozoites [11], and the pooling of samples allows screening of large samples [28]. In our study, pooling samples [11,12] and screening them by nested PCR [14,15] resulted in the identification of *P. vivax *SSUrDNA in salivary glands of two pooled *An. pseudowillmori *samples, thus establishing that *An. pseudowillmori *is the main malaria vector in this area.

## Conclusion

The composition of the local anopheline population in Motuo County at the Sino-Indian border consists mostly of members of the *An. maculatus *group, with *An. pseudowillmori *being much more abundant than *An. willmori*. *Anopheles pseudowillmori *has been identified as the local malaria vector.

## Competing interests

The authors declare that they have no competing interests.

## Authors' contributions

WS performed field and laboratory work and wrote the manuscript. PJY, WXZ and ZSS performed the field work. ZGQ, LQ performed laboratory work. TLH was involved in all stages of this study.
